# Proteomics Profiling of the Urine of Patients with Hyperthyroidism after Anti-Thyroid Treatment

**DOI:** 10.3390/molecules26071991

**Published:** 2021-04-01

**Authors:** Hicham Benabdelkamel, Afshan Masood, Aishah A. Ekhzaimy, Assim A. Alfadda

**Affiliations:** 1 Obesity Research Center, Proteomics Resource Unit, College of Medicine, King Saud University, P.O. Box 2925 (98), Riyadh 11461, Saudi Arabia; hbenabdelkamel@ksu.edu.sa (H.B.); afsmasood@ksu.edu.sa (A.M.); 2 Department of Medicine, College of Medicine and King Saud Medical City, King Saud University, P.O. Box 2925 (98), Riyadh 11461, Saudi Arabia; aishahekhzaimy@hotmail.com

**Keywords:** hyperthyroidism, urine proteomics, inflammation, acute phase proteins, apolipoproteins, carbimazole, ingenuity pathway analysis

## Abstract

Hyperthyroidism, which is characterized by increased circulating thyroid hormone levels, alters the body’s metabolic and systemic hemodynamic balance and directly influences renal function. In this study, the urinary proteome of patients with hyperthyroidism was characterized using an untargeted proteomic approach with network analysis. Urine samples were collected from nine age-matched patients before and after carbimazole treatment. Differences in the abundance of urinary proteins between hyperthyroid and euthyroid states were determined using a 2D-DIGE coupled to MALDI-TOF mass spectrometry. Alterations in the abundance of urinary proteins, analyzed via Progenesis software, revealed a statistically significant difference in abundance in a total of 40 spots corresponding to 32 proteins, 25 up and 7 down (≥1.5-fold change, ANOVA, *p* ≤ 0.05). The proteins identified in the study are known to regulate processes associated with cellular metabolism, transport, and acute phase response. The notable upregulated urinary proteins were serotransferrin, transthyretin, serum albumin, ceruloplasmin, alpha-1B-glycoprotein, syntenin-1, and glutaminyl peptide cyclotransferase, whereas the three notable downregulated proteins were plasma kallikrein, protein glutamine gamma-glutamyl transferase, and serpin B3 (SERPINB3). Bioinformatic analysis using ingenuity pathway analysis (IPA) identified the dysregulation of pathways associated with cellular compromise, inflammatory response, cellular assembly, and organization and identified the involvement of the APP and AKT signaling pathways via their interactions with interleukins as the central nodes.

## 1. Introduction

Thyroid disorders are among the commonest endocrine conditions affecting individuals worldwide. Thyroid hormones (THs), thyroxine (T4), and/or free triiodothyronine (T3) regulate many (if not all) metabolic and homeostatic processes. An increase in their circulating levels results in a hypermetabolic and catabolic clinical state known as hyperthyroidism. Numerous factors both inherent to the thyroid gland and of non-thyroidal origin are known to lead to hyperthyroidism, whose commonest cause is Grave’s disease [1]. Alterations in circulating TH levels with disease affect nearly all the organ functions and cellular metabolic processes, resulting in the generation of metabolic by-products that either directly enter the circulation or leak from tissues being ultimately filtered out by the kidney.

Systemic diseases, including thyroid diseases, are known to alter the urinary proteome (hypo urine) through the excretion of various small circulating proteins, peptides, or vesicles that pass through the glomerulus. The normal protein composition of the urine, an ultrafiltrate of the plasma, is largely made up of proteins arising from the kidney and the urinary tract, whereas a smaller fraction represents circulating proteins filtered by the glomerulus [2]. Besides the systemic and hemodynamic effects, THs influence the renal development, function, filtration capacity, and the glomerular filtration rate (GFR) of the kidney by their direct actions on it [3]. Earlier animal studies have shown that hyperthyroidism in rats led to renal hypertrophy and an increased GFR and decreased concentrating capacity of the kidney resulting in the formation of diluted urine [4,5].

Differences in proteomes between normal and disease states can be determined holistically through the use of proteomic technologies. Urine is emerging as a potential source of diagnostic biological fluid, and urine proteomics has yielded several protein non-invasive biomarkers whose identification has improved over the years but should still be thoroughly validated. Whole-expression proteomics or proteomic profiling using high-throughput proteomic technologies such as the two-dimensional difference in gel electrophoresis (2D-DIGE) is more advantageous than conventional two-dimensional polyacrylamide gel electrophoresis in comprehensively identifying them through the use of fluorescent pre-labeled proteins increasing the sensitivity, quantitative accuracy, and reducing gel-to-gel variability. Advances in proteomics have hastened the discovery of urinary biomarkers, which may lead to early diagnosis, identify risk factors, and predict the course and outcome in kidney diseases. The influence of thyroid dysfunction on altering the plasma and urinary proteome has mostly been limited to studying changes in rodent models. These studies have undoubtedly added to our understanding of TH action, although there is still a paucity of human studies. In our previous work, we used the proteomics approach in describing the alterations of the plasma proteome in patients with hyperthyroidism and hypothyroidism before and after treatment and the urinary proteome of patients with hypothyroidism [6,7,8]. In the present study, we aimed to look at the urinary proteome of the hyperthyroid patients using a quantitative 2D-DIGE followed by matrix-assisted laser desorption and ionization time of flight (MALDI-TOF) mass spectrometry (MS) during the disease state and after six months of anti-thyroid therapy in which they reverted to a normal euthyroid state.

## 2. Results

### 2.1. Anthropometric and Biochemical Data

Table 1 summarizes the parameters and biochemical data of the recruited patients. Statistically significant changes (*p* < 0.001) were observed in the biochemical profiles of FT4 (free thyroxine) and thyroid-stimulating hormone (TSH), as expected, and in the serum high-density lipoprotein levels.

### 2.2. 2D-DIGE Analysis and Identification of Differentially Expressed Proteins

**Figure 1:**
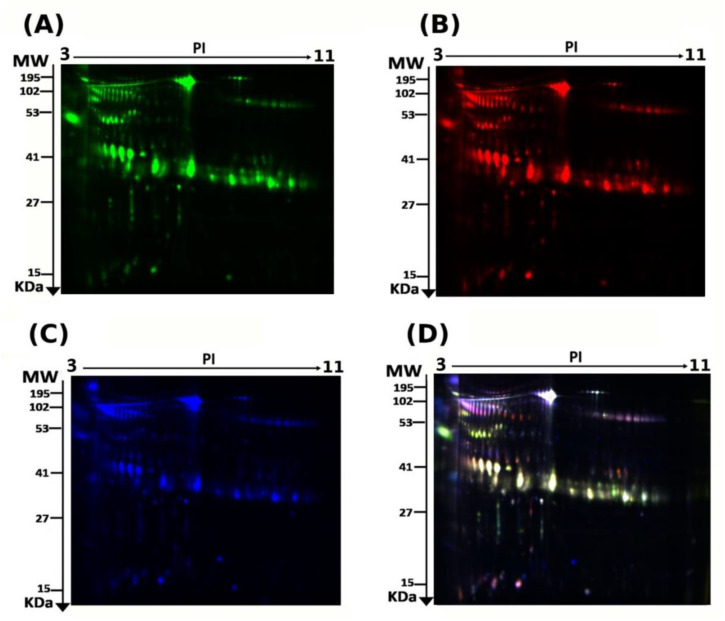
2D-DIGE analysis and identification of differentially expressed proteins. Representative fluorescent protein profiles of a 2D-DIGE containing euthyroid samples labeled with Cy3 (**A**), hyperthyroid labeled with Cy5 (**B**), and pooled internal control labeled with Cy2 (**C**), Merged 2D-DIGE comparison of Cy3/Cy5 (**D**). Urine proteins were separated on IPG strip (pH 3–11) in the first dimension followed by 12.5% PAGE in the second-dimension gel electrophoresis. Images were captured using a Typhoon 9400 Variable Mode Image.

The present study assessed the differential protein expression among nine hyperthyroid and nine euthyroid urine samples (18 samples from nine gels) through the analytical 2 dimensional—difference in gel electrophoresis (2D-DIGE) technique followed by statistical analysis using Progenesis software (Version: v3.3, Nonlinear Dynamics Ltd., UK). Cy3 labelled hyperthyroid samples with representative fluorescent protein profiles from a 2D-DIGE are displayed in Figure 1A. Hyperthyroid samples were labeled with cyanine dyes, Cy5 (Figure 1B), a pooled internal control was labeled with Cy2 (Figure 1C), and 2D-DIGE gels of samples labeled with Cy3/Cy5 overlap (Figure 1D). On the gels, there were a total of 895 spots, 40 of which were significantly different (analysis of variance (ANOVA) *p* ≤ 0.05; fold change ≥ 1.5) between the hyperthyroid and euthyroid groups. Consistency was observed for the spot patterns across all nine gels, allowing for alignment and further investigation. The internal standard, Cy2, was included to allow normalization across the entire set of gels as well as for quantitative differential analysis of protein levels. The spots (n = 40) on the preparative gel that showed statistical significance between the two groups were then manually excised for protein identification via MS.

**Figure 2:**
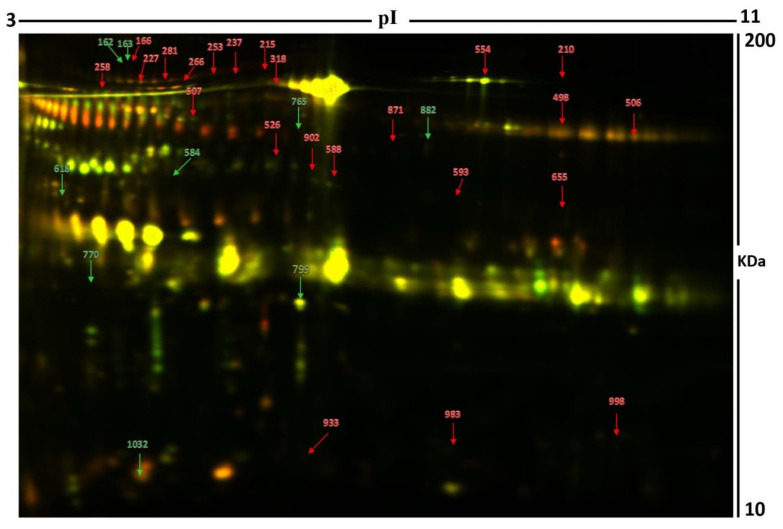
Representative image of the gel depicting the protein spots identified with MALDI-TOF (MS) in the urine samples. Numbered spots indicate proteins that were significantly changed (over 1.5-fold change, *p* < 0.05) between the hyperthyroid and euthyroid states. The red and green arrows indicate the upregulated and downregulated proteins respectively between the two states.

Using peptide mass fingerprints (PMFs), 32 out of the 40 protein spots excised from the preparative gel were successfully identified. Through MALDI-TOF MS, 26 spots were found to be unique protein sequences and were matched to entries in the SWISS-PROT database via Mascot with high confidence scores (Figure 2, Table 2, Supplementary Table S2). The sequence coverage of the proteins identified by PMF ranged from 4% to 81%. In a few cases, variants of the same protein were found at several locations on the gel (Table 2, Figure 2). Among the 32 proteins identified, 26 protein spots were upregulated, and seven were downregulated in the samples of patients with hyperthyroidism in comparison with that in the euthyroid subjects (Table 2, Figure 2). The significantly upregulated proteins included protein S100-A9 (up 3.4-fold, *p* = 0.049), phosphatidylinositol 4-phosphate 3-kinase C2 domain-containing subunit alpha (up 3.2-fold, *p* = 0.034), serpin B3 (up 3.0-fold, *p* = 0.009), keratin, type I cytoskeletal 10 (up 2.6-fold, *p* = 0.028), transthyretin (up 2.5-fold, *p* = 0.004), serum albumin (up 2.1-fold, *p* = 0.006), alpha-1B-glycoprotein (up 2.0-fold, *p* = 9.410–4), syntenin-1 (up 1.8-fold, *p* = 0.016), glutaminyl-peptide cyclotransferase (up 1.8-fold, *p* = 0.01), and polymeric immunoglobulin receptor (up 1.7-fold, *p* = 0.017) (complete list provided in Table 2). The significantly downregulated proteins in hyperthyroid/euthyroid state included plasma kallikrein (down 2.5-fold, *p* = 0.023), protein glutamine gamma-glutamyl transferase Z (Glu-GGT) (down 1.6-fold, *p* = 0.031), zinc finger protein 322 (down 2.5-fold, *p* = 0.029), serine/threonine-protein phosphatase 2A activator (PTPA) (down 2.0-fold, *p* = 0.03), SH3 domain-binding glutamic acid-rich-like protein 3 (down 3.2-fold, *p* = 0.02), polymeric immunoglobulin receptor (down 1.9-fold, *p* = 0.054), and tRNA-specific adenosine deaminase 1 (down 1.5-fold, *p* = 0.051) (Table 2, Supplementary Table S2). Among the identified proteins, alpha-1B-glycoprotein, serum albumin, glutaminyl-peptide cyclotransferase, and polymeric immunoglobulin receptors were found in more than one spot on the gels, which could be explained by posttranslational modifications, cleavage by enzymes, or the presence of different protein species.

### 2.3. Principal Component Analysis

**Figure 3:**
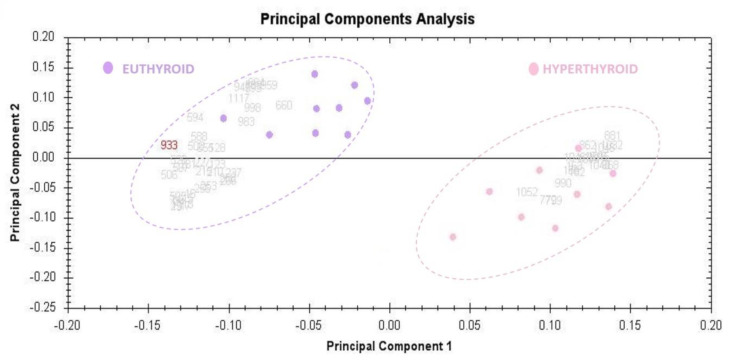
The Progenesis SameSpots software was used to assess and visualize the samples of hyperthyroid and euthyroid subjects using the principal component analysis (PCA). All 40 spots with statistically significant (ANOVA, *p* < 0.05) changes in abundance determined by MS were subjected to PCA. The tests showed that the two groups clustered differently based on different proteins, with a significant score of 62 percent.

The principal component analysis (PCA) was performed using Progenesis SameSpots software (Version: v3.3, UK) to determine and visualize the samples of the hyperthyroid and euthyroid subjects. The PCA was performed on all 40 spots that exhibited statistically significant (ANOVA, *p* < 0.05) changes in abundance as identified via MS. The analyses revealed that the two groups clustered distinctly from one another based on different proteins, with 62% as a significant score (Figure 3).

### 2.4. Network Pathway Analysis

**Figure 4:**
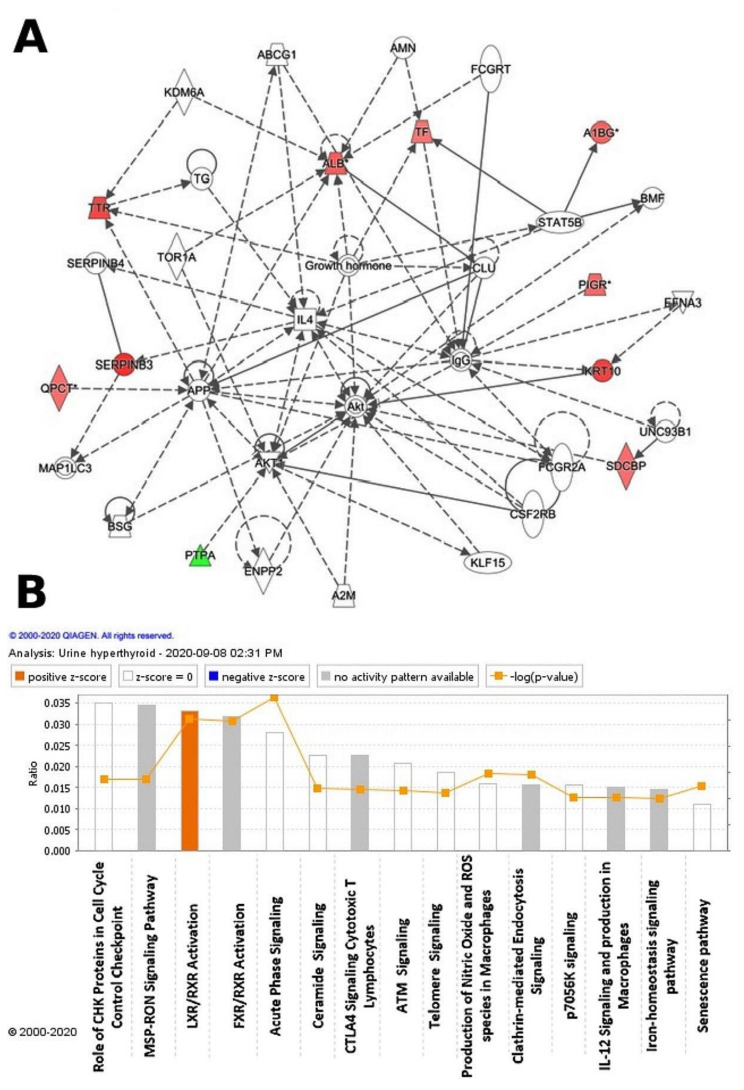
The differentially expressed proteins with their most enriched interaction network in the hyperthyroid state compared with the euthyroid state. Upregulated nodes are red, while downregulated nodes are green. Between the two states, the central nodes of the pathway related to signaling of the ALB, IL4, APP, AKT1, Akt, and IgG were found to be deregulated. IPA proposes uncolored nodes that represent possible targets that were functionally coordinated with the differentially expressed proteins. Direct molecular interactions are indicated by solid lines, while indirect molecular interactions are indicated by dotted lines (**A**). The top 15 canonical pathways, ranked by *p*-values obtained via the IPA, are depicted in this diagram (**B**).

Ingenuity pathway analysis (IPA) was used to assess the 32 differentially regulated proteins for protein–protein interactions. The analysis found that 10 proteins interacted directly or indirectly through protein networks, out of a total of 32 proteins (Figure 4A). To create a protein–protein interaction network, the program computes a score based on the best fit obtained from the input data set of proteins and the biological functions database. The resulting network is enriched for proteins with complex and extensive interactions, with interacting proteins represented as nodes and their biological relationships represented as a line. Three interaction networks were discovered based on the data for the proteins with different expression profiles. The network with the highest score (score = 20) (Figure 4, Supplementary Figure S2) included 21 proteins. The proposed network pathway with the highest number of interactions was associated with cellular compromise and inflammatory response, cellular assembly, and organization. Only the top pathways are shown (Figure 4A). Figure 4B shows canonical pathways enriched in the current dataset. The canonical pathways in Figure 4B are sorted down to decreasing log (*p*-value) of enrichment. The three topmost enriched canonical pathways included acute phase response signaling (2.8% overlap, *p*-value: 1.34 × 10^−6^), liver X receptors/retinoid X receptor (LXR/RXR) activation (3.3% overlap, *p*-value: 8.86 × 10^−6^), farnesoid X receptors/retinoid X receptor (FXR/RXR) activation (3.2% overlap, *p*-value: 1.04 × 10^−6^), clathrin-mediated endocytosis signaling (1.6% overlap, *p*-value: 1.21 × 10^−3^), and production of nitric oxide and reactive oxygen species in macrophages (1.6% overlap, *p*-value: 1.21 × 10^−3^). Supplementary Figure S2 summarizes the details of the canonical pathways identified in this study.

### 2.5. Function Analysis

**Figure 5:**
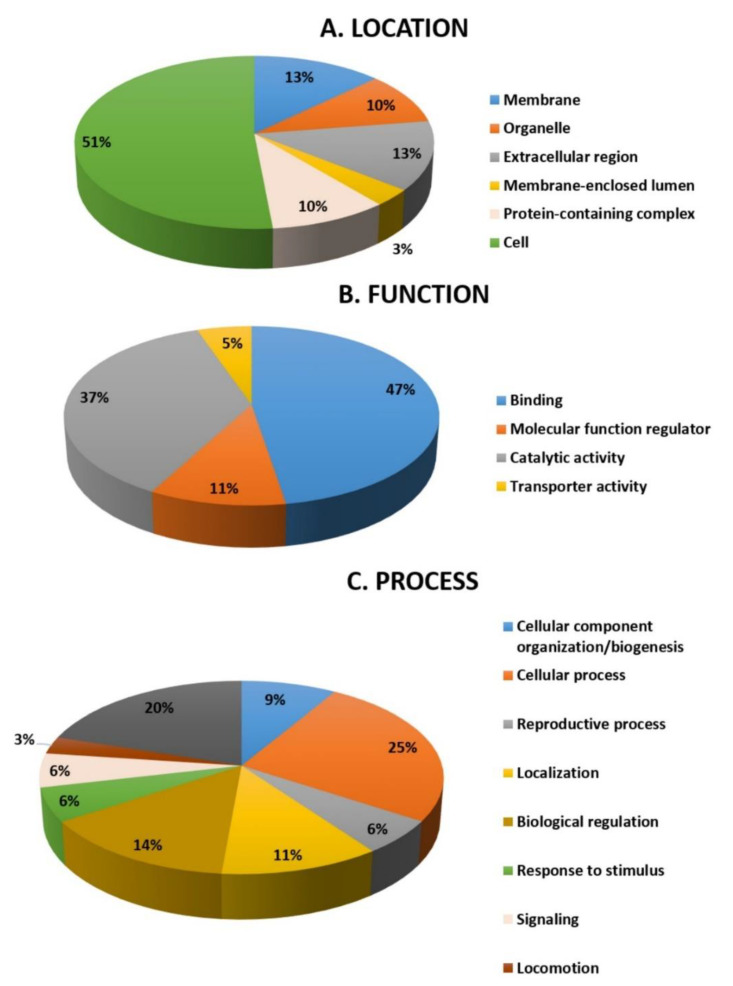
Comparative depiction (%) of the significantly identified proteins using MALDI-TOF (MS): categorized into groups according to their location (**A**), function (**B**), and process (**C**).

All 32 differentially abundant proteins found between the hyperthyroid and euthyroid samples were subjected to the PANTHER (protein analysis through evolutionary relationships) classification system (http://www.pantherdb.org accessed on 29 March 2021) after MS for classification according to their function (Figure 5A), location (Figure 5B), and process (Figure 5C). As shown in Figure 5A, the most common functional categories found were binding proteins (35%), molecular functional regulators (23%), and proteins involved in catalytic activity (42%). Furthermore, the majority of the proteins identified were found in the cellular part (51%), followed by the membrane (13%), extracellular region (13%), organelle (10%), the protein-containing complex (10%), and membrane closed lumen (3%) as shown in Figure 5B. The majority of the identified proteins were involved in cellular processes and a few in transportation Figure 5C.

### 2.6. Immunoblotting Confirmation of Changes in Selected Proteins

**Figure 6:**
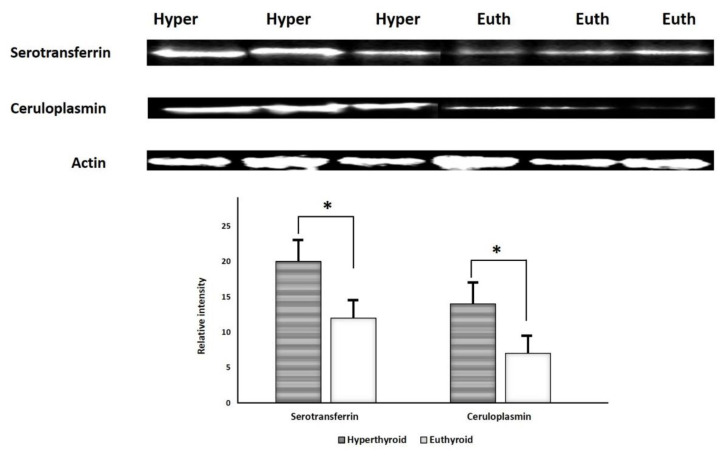
Immunoblot analysis of selected proteins identified by 2D-DIGE analysis was used to validate the proteomic results. The findings of immunoblotting were similar to the results of 2D-DIGE (**A**). Graphical representation of the relative intensity values of normalized protein bands between the hyperthyroid and euthyroid states. The data are reported as histograms of the mean ± SD (**B**). The * indicates *p* < 0.05.

Immunoblot analysis successfully validated key proteins that were differentially abundant between the groups (Figure 6). The proteins targeted for confirmation included: serotransferrin and ceruloplasmin. Immunoblots confirmed a significantly (*p* ≤ 0.05) different expression of these proteins in hyperthyroid urine samples compared to euthyroid urine samples. The housekeeping protein β-actin was used to normalize the immunoblot results (Figure 6A,B).

## 3. Discussion and Conclusions

Hyperthyroidism is a dysfunction of the thyroid gland characterized by an increase in the levels of circulating free TH. Clinically, patients with hyperthyroidism present with palpitations, fatigue or weakness, anxiety, tremors, nervousness, mood swings, increased sensitivity to heat, and thinning skin [9]. Urine is an easily acquired biological fluid and measurement of urinary protein serves as a non-invasive method for detection of markers for clinical diagnosis. The normal protein concentration of the healthy urine is very low (less than 100 mg/L), with protein excretion of less than 30 mg/day. An excretion of more than 30 mg/day of protein is considered as proteinuria and is indicative of systemic disease affecting the kidney [10]. Proteomics of the urine was considered for analysis as urinary proteome has a less complex matrix for analysis of proteins than plasma. Although true, this view has changed since a large number of proteins (within the normal clinical range) have been identified in the healthy urine proteome [11] using two different separation techniques, one-dimensional sodium dodecyl sulfate–polyacrylamide gel electrophoresis (SDS-PAGE) and reverse-phase high-performance liquid chromatography for protein separation and fractionation. The proteins identified in the normal urinary proteome are mainly extracellular in origin, followed by those derived from the plasma membrane, extracellular vesicles, and intracellular organelles (nucleus and lysosomes) and are functionally associated with signal transduction, peptidase activity, immune response proteins, protease inhibitors, adhesion molecules, and cytokines [12]. The urinary proteins identified in the present study are functionally (i) acute phase proteins, (ii) transport proteins, (iii) enzymes, and (iv) transmembrane domain proteins.

### 3.1. Proteins Increased in the Urine of Patients with Hyperthyroidism

In the present study, 25 protein spots corresponding to unique proteins were identified. Among these proteins, four proteins, serotransferrin, ceruloplasmin, transthyretin, and serum albumin, are known acute phase proteins involved in the acute phase inflammatory response in conditions of increased chronic inflammation [13]. These proteins are also known to function as plasma proteins involved in transport and the chronic inflammatory process. The levels of these proteins were observed to be raised in both hyperthyroidism and hypothyroidism, indicating that both diseases had an underlying state of chronic inflammation. In addition to these, we also identified an increase in transthyretin and ceruloplasmin that also participate in the APR.

An interesting protein identified with a significant increase in abundance in the urine samples obtained from patients with hyperthyroidism is protein S100-A9. Protein S100-A9 belongs to a family of calcium-binding phagocyte-specific proteins that exhibit diverse functions and cell-specific expression patterns. It is known to exist as a heterodimer in conjunction with S100A8 (S100A8/A9 or calprotectin) that is secreted in response to inflammation [8] and has emerged as a reliable biomarker of disease activity with diagnostic, prognostic, and predictive potential [14]. Increased levels of S100A8/A9 have been observed in a multitude of inflammatory diseases, including adult SLE, systemic juvenile idiopathic arthritis, and inflammatory bowel disease, in predicting the development of chronic kidney disease and distinguishing between intrinsic and prerenal acute kidney injury [15]. S100 proteins have particularly been linked to thyroid tumorigenesis and metastasis; increased S100A4 expression in papillary thyroid carcinoma, whereas proteins S100-A10 and S100-A6 have been identified as biomarkers of papillary thyroid carcinoma [16] with lymph node metastasis [17]. Protein S-100 expression was found by Nishimura et al. to be present in the thyroid follicular cells, correlated with degree of thyroglobulin synthesis and that its level was increased in hyperfunctional states and decreased in hypofunctional states [18]. The identification of protein S100–A9 in the urine of patients with hyperthyroidism is being reported for the first time. To identify the role of this protein in noncancerous thyroid disease, more mechanistic studies are required.

A member of the immunoglobulin superfamily, α-1glycoprotein, was identified to have diverse functions from protein binding to cell adhesion and in molecular recognition in the immune system. Previous studies using animal models have shown that aging, sex, and growth hormones contribute to its differential expression [19]. The structure and chromosomal allocation of α-1glycoprotein hve been known for over three decades, although its associations with disease are known with only a few [20]. We previously reported increased levels of the protein in the plasma proteome of patients with hyperthyroidism [20] and observed in the urine of patients with steroid-resistant nephrotic syndrome and bladder cancer in the pancreatic juice of patients with pancreatic ductal adenocarcinoma in cell lines of liver cancer [21,22,23,24].

Serpin B3 is a serine proteinase inhibitor acting on cysteine proteinases such as cathepsin and chymotrypsin. Several in vitro and in vivo studies have documented an important role of SERPIN B3 in modulating programmed cell death by different mechanisms [25] for a role of SERPIN B3 in inhibiting inflammation and favoring increased epithelial proliferation and fibrosis [25], which could subsequently lead to the development of renal tubulointerstitial fibrosis and chronic kidney disease. An increase of SERPIN B3 in the hyperthyroid state might correspond to a compensatory mechanism to counteract the increased inflammatory state.

We identified an increase in the protein spots relating to Syntenin-1 in the urine of patients in the hyperthyroid state in comparison with the euthyroid state. Syntenin-1 is an adapter protein that belongs to the family of PDZ-domain-containing proteins, which acts as a scaffolding protein for the binding of numerous signaling proteins and receptors. It plays an essential role in modulating signal transduction from the extracellular to the intracellular environment through numerous protein–protein interactions, thereby controlling diverse and central physiologic processes [26]. It is an important component at cell adhesion sites and has also been identified to localize predominantly in the early secretory pathway, such as the endoplasmic reticulum, and forms a major structural component of apical early endosomes, facilitating the trafficking of cell-surface-located molecules. It is known to be involved in the organization of protein complexes in plasma membranes, regulation of B-cell development, intracellular trafficking and cell-surface targeting, synaptic transmission, and axonal outgrowth [27]. Koo et al. demonstrated an increase in the levels of syntenin-1 in rapidly migrating and metastasizing breast and gastric cancer cells [28]. Syntenin-1 is known to be involved in the trafficking of receptor-type tyrosine phosphatase, whose expression was found to be reduced in several thyroid oncogene-transformed cells or absent in highly malignant thyroid cells [28].

Along with syntenin-1, another transmembrane domain protein identified in our study was PI4phosphate 3kinase (PI4P3K) domain protein. The PI4P3K proteins are known to interact with syntenin proteins [29]. The PI4P3K protein belongs to a large group of tetraspanin proteins implicated in a variety of biological phenomena, including wound healing, immune responses, tumor cell migration, and invasion [25,30], maturation and processing of associated transmembrane proteins, and intracellular sorting of the associated receptors [31].

### 3.2. Proteins Decreased in the Patients with Hyperthyroidism

Seven proteins were found to be significantly decreased in abundance in the urine of patients in the hyperthyroid state in comparison with those in the euthyroid state. Three notable proteins identified among the seven were plasma kallikrein and PTPA and protein glutamine gamma-glutamyl transferase.

Plasma kallikrein is another serine protease identified in our study, although with a decreased abundance. It belongs to the kinin–kallikrein system (KKS) that liberates kinins and vasoactive peptides (bradykinin and kallidin) from their proform kininogens. Plasma kallikrein plays a central role in regulating the renin–angiotensin–aldosterone system (RAAS) and the KKS. The KKS-RAAS system is a complex system involved in the regulation of inflammation, blood pressure, cardiovascular function, vascular tone, hemostasis, and pain. The KKS in the kidney plays a significant role in the regulation of renal blood flow and water-electrolyte balance and largely counterbalances the RAAS. The balance between RAAS and KKS affects BP, salt sensitivity, circulating volume, and vascular tone. Increased amounts of kinins released by the action of plasma kallikrein lead to vasodilatation and reduce blood pressure and may be involved in the pathogenesis of hypertension and renal failure [32]. Our findings are in contrast to those of Avigdor et al., who showed that urine kallikreins were decreased in the thyroidectomized rats and increased with thyroxine treatment [33], whereas decreased urinary kallikrein excretion was seen in both humans and rodents with HTN [34]. Besides these actions, plasma kallikrein is involved in regulating hemostasis, and along with its product bradykinin, can generate plasmin from plasminogen, allowing fibrinolysis to occur. A decrease in the levels of plasma kallikrein excretion might reflect decreased synthesis. This could lead to a disruption of the normal hemostasis due to a decreased fibrinolytic activity in blood, predisposing patients with hyperthyroidism to experience vascular endothelial dysfunction and represent a situation with a higher thromboembolic potential [35].

Protein glutamine gamma-glutamyl transferase, an enzyme of the transglutaminase family that catalyzes the posttranslational modification of proteins at glutamine residues, with the formation of isopeptide bonds, cross-linking of proteins, and the conjugation of polyamines to proteins [36]. The levels of this enzyme were observed by our group to be decreased in both the hypothyroid and the hyperthyroid states. THs are known to regulate amino acids, including glutamine metabolism [37], although the role of this enzyme with alterations in the levels of TH requires further study.

We identified a decrease in the abundance of serine/threonine-protein phosphatase activator (also known as phosphotyrosyl phosphatase activator (PTPA)), which is responsible for stimulating the phosphotyrosyl phosphatase (PTPase), such as protein phosphatase 2A, which catalyzes the reversible dephosphorylation of tyrosyl residues of their substrates that include the receptor tyrosine kinases to promote protein–protein interactions within the cell. Protein phosphatase 2A is a major cytoplasmic serine/threonine phosphatase that plays an essential role in the regulation of cell growth and a diverse set of cellular proteins, including metabolic enzymes, ion channels, hormone receptors, and kinase cascades [38]. Reversible tyrosine phosphorylation is one of the most important posttranslational modifications that regulate key aspects of cellular biology such as protein stability, protein–protein interactions, and enzyme activity, thereby modulating the functionality of fundamental elements involved in signaling transduction of mammalian cells [39]. Cytoplasmic Shp-2 plays a crucial role in receptor-activated pathways in insulin gene transcription, modulating signals that flow through PI3K/Akt/FoxO1 and extracellular signal-related kinase pathways and culminates in the control of the genomic activity. PTPA inactivates and negatively regulates AKT, a subfamily of serine/threonine-protein kinases that regulate a variety of cellular processes during normal tissue homeostasis and cell transformation signaling by dephosphorylation [40]. The involvement and dysregulation of the AKT pathway between the hypothyroid and euthyroid states were also shown to be the central nodes with the highest connectivity in the network pathway identified by network pathway analysis.

## 4. Materials and Methods

### 4.1. Ethical Considerations and Informed Consent

All procedures and protocols, including clinical samples, were reviewed and approved by the Institutional Review Board of the College of Medicine, King Saud University, Riyadh, Saudi Arabia. The study was conducted following the ethical standards of the Declaration of Helsinki and the universal ICH-GCP guidelines.

### 4.2. Study Design

Nine individuals with newly diagnosed overt hyperthyroidism were referred to our endocrine outpatient clinic at the King Khaled University Hospital, with an average age of 39.6 ± 10.6 years. After fasting for 10 h, blood samples were taken from each patient before (pre-treatment sample) and after (post-treatment sample) beginning anti-thyroid medication. To determine the minimum number of necessary biological replicates, a power analysis was performed using the Progenesis SameSpots nonlinear dynamics statistical program (Supplementary Figure S1). FT4 (free thyroxine) levels greater than 22 pmol/L and TSH levels less than 0.25 mIU/L were used to define hyperthyroidism. Pre-treatment samples (hyperthyroid state) were collected before beginning anti-thyroid therapy, and post-treatment samples (euthyroid state) were collected from patients with normalized FT4 levels after treatment with the appropriate dose of anti-thyroid medication (carbimazole). There was no history of hypertension, type 2 diabetes, or other inflammatory or autoimmune disorders among patients in the study. Venipuncture into EDTA-coated tubes was used to extract blood samples, and plasma was obtained by centrifugation (15 min, 3000× *g*), which was then stored in several aliquots at −80 °C before analysis.

### 4.3. Protein Extraction from Urine Samples

After a standard 10 h fast, midstream spot urine samples (50–100 mL) were collected in a clean-catch specimen into a sterile urine container and immediately transported in ice to avoid microbial contamination and proteolysis. We used urine test strips to screen for urinary protein, urinary infection, urinary sugar, and occult blood (Combur10Test, Roche: Basel, Switzerland). Within 30 min of collection, the samples were processed, and insoluble materials were removed by centrifugation at 2000× *g* (4000 rpm) at 4 °C for 10 min to avoid protein release from these artifacts. For long-term storage, the supernatants were carefully removed and frozen in 2 mL aliquots at −80 °C. Proteins were extracted from urine samples as previously reported [41]. Centrifugation (12,000× *g*, room temperature, 5 min) was used to pellet insoluble material. In a labeling buffer, the pellets were solubilized (7 M urea, 2 M thiourea, 30 mM Tris-HCl, 4% CHAPS, pH 8.5). The 2D-Quant Kit was then used to determine the concentration of protein samples in triplicate (GE Healthcare, Chicago, IL, USA).

### 4.4. Fluorescence Labeling, 2D-DIGE, and MALDI-TOF-MS Analysis

DIGE analysis was used in determining the differentially expressed proteins between the hyperthyroid vs. euthyroid groups as described [7]. Each sample of the hyperthyroid and euthyroid groups had around 50 ug of protein labeled with 400 pmol of Cy3 and Cy5 dyes. As an internal standard, a mixture of an equal quantity of all samples was pooled, labeled with Cy2, and used. To prevent dye-specific bias, a dye switching technique was used during labeling (Supplementary Table S1). First-dimension analytical gel electrophoresis, followed by second-dimension SDS-PAGE, was performed on 12.5% fixed gels as previously described [7].

Additionally, the 2D-DIGE gels were scanned in a Typhoon 9410 scanner at specific excitation/emission wavelengths for Cy2 (488/520 nm), Cy3 (532/580 nm), and Cy5 (633/670 nm). The 2D-DIGE gel images were uploaded into the Progenesis SameSpots program (Nonlinear Dynamics Ltd., NE1 2JE, UK) and an automated spot detection system was used to analyze them. A comparison of hyperthyroid and euthyroid samples was included in the analysis. Despite the fact that the automated analysis detected all of the spots across all nine gels, each selected spot was manually verified and edited as required. To find spots that were differentially expressed, normalized volumes were used. A 1.5-fold cut-off ratio was considered statistically important. The fold difference and *p*-values were calculated using a one-way ANOVA.

The preparatory gel with Coomassie-stained gel spots was washed and digested as mentioned previously [7,41]. A MALDI target (384 MTP Anchorchip; 800 m Anchorchip; Bruker Daltonics, Bremen, Germany) was spotted with a mixture of tryptic peptides (1 uL) derived from each protein. As previously mentioned [7,42,43], MALDI-TOF (MS) spectra were obtained using an UltraflexTerm TOF mass spectrometer equipped with a LIFT-MS/MS device (Bruker Daltonics) at reflector and detector voltages of 21 and 17 kV, respectively. Using Flex Analysis software, the PMFs were assessed (version 2.4, Bruker Daltonics). BioTools v3.2 was used to interpret MS data (Bruker Daltonics). The Mascot search algorithm (v2.0.04, updated on 09/05/2019; Matrix Science Ltd., London, UK) was used to search the peptide masses. Mascot parameters were as follows: fixed cysteine modification with propionamide, variable modification due to methionine oxidation, one missed cleavage site (i.e., in the case of incomplete trypsin hydrolysis), and amass tolerance of 100 ppm. Identified proteins were accepted as correct if they showed a Mascot score greater than 56 and *p* < 0.05.

### 4.5. Bioinformatic Analysis: Functional Classification of Proteins and Pathway Analysis

The proteins that were successfully identified were then analyzed using the IPA Software program (Ingenuity^®^ Systems, version: 2000–2020, http://www.ingenuity.com accessed on 29 March 2021, California, USA) to determine their functions and pathways. Based on previous publications on the proteins, the annotations included overlaying the proteins with their most important networks and biochemical pathways. Using the PANTHER classification system (http://www.pantherdb.org accessed on 29 March 2021), the identified proteins were further categorized into different categories based on their function, location, and processes.

### 4.6. Statistical Analysis

The results on the biochemical parameters in the hyperthyroid and euthyroid groups were presented as mean ± SD, and significant differences between the mean values were assessed using Student’s test. A PCA of the log-transformed spot data was performed.

### 4.7. Immunoblotting

To independently validate the results of the 2D-DIGE analysis, immunoblotting was used to test statistically important proteins with differential abundance. Monoclonal antibodies against transferrin (mouse, cat # SC365871-), ceruloplasmin (mouse, cat # SC-365206), and β-actin (goat, N-18, cat # SC-1616), were purchased from Santa Cruz Bio-technology (Santa Cruz, CA, USA). One-dimensional discontinuous slab gel electrophoresis (12% sodium dodecyl sulfate (SDS)-polyacrylamide gel) was used to separate an equal amount of protein from each sample (50 μg). Proteins were electrotransferred to an Immobilon-P, poly(vinylidene difluoride) (PVDF) transfer membrane (Millipore) using a mini trans-blot electrotransfer cell (BioRad). The membrane was stained with Ponceau-S after the transfer to confirm the transfer efficiency. The membrane was then blocked (5% fat-free milk (FFM) in trisbuffered saline (TBS), 1 h, RT), and rinsed (three changes of TBS-T in 10 mM Tris−HCl, 150 mM NaCl, 0.1% Tween 20 buffer). In a blocking buffer, the samples were incubated with the specified primary antibodies (1:200 dilution). Blots were incubated with the appropriate immunoglobulin G (IgG)-horseradish peroxidase (HRP)-conjugated secondary antibody, immunoreactive bands were detected using enhanced chemiluminescence (ECL, Thermo Scientific, Waltham, MA, USA), visualized using Fluorchem Q (Cell Biosciences, Santa Carla, USA), and digitalized using the image analysis software AlphaView Q 3.0 (Cell Biosciences, Santa Carla, CA, USA).

## Figures and Tables

**Table 1:** Biochemical parameters of the hyperthyroid study subjects before and after carbimazole therapy. FT4, free thyroxine; TSH, thyroid-stimulating hormone; HDL, high-density lipoprotein; LDL, low-density lipoprotein.

	Normal Range	Hyperthyroid	Euthyroid	*p*-Value
N		9	9	
Age (years)		39.6 ± 10.6	39.6 ± 10.6	
Glucose (mmol/L)	4.07–5.83	5.3 ± 0.8	5.2 ± 0.5	0.19
Urea (mmol/L)	2.5–6.4	3.9 ± 0.9	4.6 ± 0.8	0.18
Creatinine (µmol/L)	53–115	61.1 ± 12.7	66.3 ± 12.0	0.39
Sodium (mmol/L)	136–145	138.6 ± 2.1	138 ± 0.9	0.16
Potassium (mmol/L)	3.5–5.1	4.2 ± 0.2	4.3 ± 0.4	0.40
Aspartate transaminase (IU/L)	15–37	34.8 ± 9.1	31.8 ± 4.5	0.38
Alanine transaminase (IU/L)	20–65	17.5 ± 5	15.8 ± 2.0	0.40
Alkaline phosphatase (IU/L)	40–150	114.6 ± 53.7	120.2 ± 28.9	0.30
FT4 (pmol/L)	11.5–22.7	35.4 ± 9.9	17.0 ± 2.8	0.001
TSH (mIU/L)	0.25–0.5	0.014 ± 0.01	0.8 ± 0.4	0.00068
Total cholesterol (mmol/L)	3.2–5.2	4.6 ± 1.0	4.9 ± 0.8	0.10
HDL cholesterol (mmol/L)	0.96–2.15	2.8 ± 0.9	3.1 ± 0.7	0.02
LDL cholesterol (mmol/L)	1.84–4.25	2.8 ± 0.9	3.1 ± 0.7	0.09
Triglycerides (mmol/L)	0.4–1.48	1.1 ± 0.6	0.9 ± 0.2	0.14

**Table 2:** Identified proteins, with changes in abundance of significantly differentially abundant proteins between hyperthyroid and euthyroid states in urine samples. Table 2 shows values for the average ratio between the two states, with their corresponding levels of fold changes and one-way ANOVA (*p*-value < 0.05) using 2D-DIGE. (Analysis type: MALDI-TOF; database: SwissProt; taxonomy: Homo sapiens).

Sl No	Spot No.	Accession No. ^a^	Protein Name	MASCOT ID	*p*-Value ^b^ (ANOVA)	Ratio ^c^ Hyper\Euth	EXP ^d^
1	554	P02787	Serotransferrin	TRFE_HUMAN	0.048	1.7	UP
2	871	P06702	Protein S100-A9	S10A9_HUMAN	0.049	3.4	UP
3	933	P04217	Alpha-1B-glycoprotein	A1BG_HUMAN	9.410^−4^	2	UP
4	227	P02768	Serum albumin	ALBU_HUMAN	0.006	2.1	UP
5	210	Q6ZMW3	Echinoderm microtubule-associated protein-like 6	EMAL6_HUMAN	0.001	1.6	UP
6	983	P02766	Transthyretin	TTHY_HUMAN	0.004	2.5	UP
7	253	P02768	Serum albumin	ALBU_HUMAN	0.017	1.6	UP
8	237	P01833	Polymeric immunoglobulin receptor	PIGR_HUMAN	0.017	1.7	UP
9	258	P04217	Alpha-1B-glycoprotein	A1BG_HUMAN	0.024	1.6	UP
10	166	P00450	Ceruloplasmin	CERU_HUMAN	0.029	1.8	UP
11	588	Q9UQ35	Serine/arginine repetitive matrix protein 2	SRRM2_HUMAN	0.031	1.7	UP
12	593	O00443	Phosphatidylinositol 4-phosphate 3-kinase C2 domain-containing subunit alpha	P3C2A_HUMAN	0.034	3.2	UP
13	318	P02768	Serum albumin	ALBU_HUMAN	0.036	1.6	UP
14	526	Q16769	Glutaminyl-peptide cyclotransferase	QPCT_HUMAN	0.01	1.8	UP
15	215	Q6FIF0	AN1-type zinc finger protein 6	ZFAN6_HUMAN	0.02	1.6	UP
16	266	Q92878	DNA repair protein RAD50	RAD50_HUMAN	0.047	1.5	UP
17	584	Q9BUB4	tRNA-specific adenosine deaminase 1	ADAT1_HUMAN	0.051	−1.5	DOWN
18	162	P03952	Plasma kallikrein	KLKB1_HUMAN	0.023	−2.5	DOWN
19	618	Q15257	Serine/threonine-protein phosphatase 2A activator	PTPA_HUMAN	0.03	−2.0	DOWN
20	163	Q96PF1	Protein glutamine gamma-glutamyl transferase Z	TGM7_HUMAN	0.031	−1.6	DOWN
21	799	Q6U7Q0	Zinc finger protein 322	ZN322_HUMAN	0.029	−2.5	DOWN
22	882	P29508	SERPINB3	SPB3_HUMAN	0.009	3	UP
23	765	Q9UKF7	Cytoplasmic phosphatidylinositol transfer protein 1	PITC1_HUMAN	0.031	1.7	UP
24	902	Q16769	Glutaminyl-peptide cyclotransferase	QPCT_HUMAN	0.048	1.5	UP
25	281	P01833	Polymeric immunoglobulin receptor	PIGR_HUMAN	0.011	1.7	UP
26	998	Q8N3U1	Putative uncharacterized protein LOC400692	YS014_HUMAN	0.011	1.5	UP
27	506	Q4V348	Zinc finger protein 658B	Z658B_HUMAN	0.016	1.5	UP
28	655	O00560	Syntenin-1	SDCB1_HUMAN	0.016	1.8	UP
29	1032	Q9H299	SH3 domain-binding glutamic acid-rich-like protein 3	SH3L3_HUMAN	0.02	−3.2	DOWN
30	959	P13645	Keratin, type I cytoskeletal 10	K1C10_HUMAN	0.028	2.6	UP
31	770	P01833	Polymeric immunoglobulin receptor	PIGR_HUMAN	0.054	−1.9	DOWN
32	498	P04746	Pancreatic alpha-amylase	AMYP_HUMAN	0.052	1.5	UP

^a^ Protein accession number for SWISSPROT Database; ^b^ *p*-Value (ANOVA); ^c^ ratio between the groups; ^d^ protein expression between the groups.

## Data Availability

All data generated or analyzed in the current study are included in this article.

## Supplementary Materials

The following are available online, Figure S1: Power calculation, Figure S2: Canonical pathways, Table S1: Experimental design, Table S2: Mass spectrometry list of significant differentially abundant proteins.
