# Supplementary material for: Proteomics Profiling of the Urine of Patients with Hyperthyroidism after Anti-Thyroid Treatment
Source: Molecules. 2021 Apr 1;26(7):1991. doi: 10.3390/molecules26071991 (PMC8036843; doi:10.3390/molecules26071991)
Supplement: Supplementary file 1 [file molecules-26-01991-s001.pdf]

Supplementary material

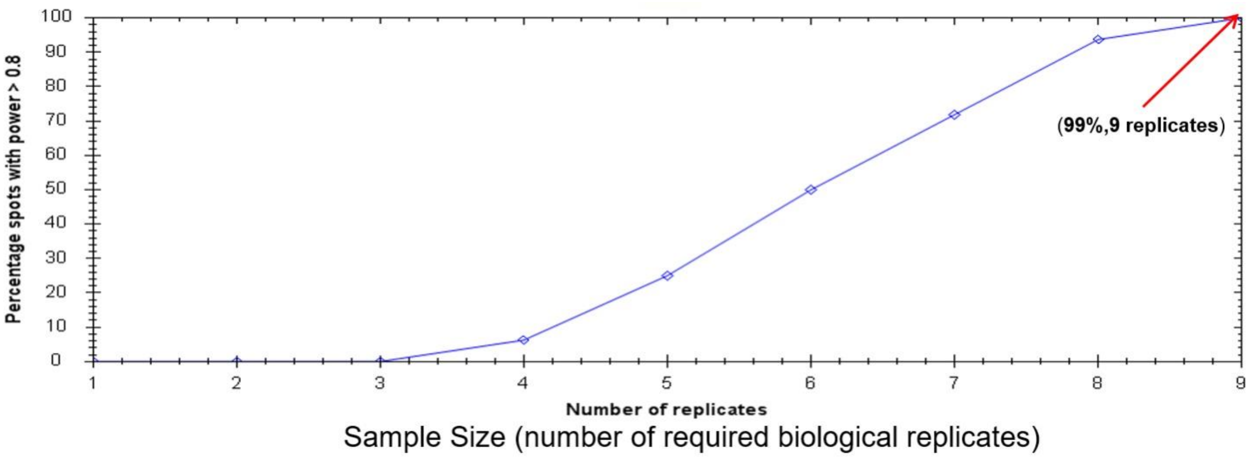

Figure S1: Power calculation for determination of the minimum number of required biological variants for 2-DIGE analysis. The power curve was used to calculate the sample size required to find significant difference with a fold-change of ≥1.5 between two paired groups at 98% power and p-value ≤ 0.05.

Figure S2: The figure shows the different canonical pathways obtained from IPA functional analysis.

Top Canonical Pathways

Name	p-value	Overlap
Acute Phase Response Signaling	1.34E-06	2.8 % 5/179
LXR/RXR Activation	8.86E-06	3.3 % 4/121
FXR/RXR Activation	1.04E-05	3.2 % 4/126
Production of Nitric Oxide and Reactive Oxygen Species in Macrophages	1.12E-03	1.6 % 3/188
Clathrin-mediated Endocytosis Signaling	1.21E-03	1.6 % 3/193

Table S1: Experimental design: 18 samples run on 9 2D-PAGE gels, samples were labeled randomly with Cy3 and Cy5, and a pooled sample was used as an internal standard and was stained with Cy2

Cy2	Cy5	Cy3	Gel
Pooled sample	11<br>(hyperthyroid)	1<br>(Euthyroid)	1
Pooled sample	2<br>(Euthyroid)	12<br>(hyperthyroid)	2
Pooled sample	13<br>(hyperthyroid)	5<br>(Euthyroid)	3
Pooled sample	3<br>(Euthyroid)	14<br>(hyperthyroid)	4
Pooled sample	6<br>(Euthyroid)	16<br>(hyperthyroid)	5
Pooled sample	17<br>(hyperthyroid)	7<br>(Euthyroid)	6
Pooled sample	8<br>(Euthyroid)	18<br>(hyperthyroid)	7
Pooled sample	19<br>(hyperthyroid)	9<br>(Euthyroid)	8
Pooled sample	10<br>(Euthyroid)	20<br>(hyperthyroid)	9

Table S2: Mass spectrometry list of significant differentially abundant proteins between Hyper and Euth identified in urine samples, using 2D-DIGE with. Protein name, accession number, Mascot score, MS % coverage, protein MW and pI values according to Uniprot database are listed.

Sl no	SpotNo	Accession No[a]	Protein Name	MASCOT ID	Pi[b]	MW[c]	Cov%	Score[d]
1.	554	P02787	**Serotransferrin**	TRFE_HUMAN	6.81	74280	39	104
2.	871	P06702	**Protein S100-A9**	S10A9_HUMAN	5.71	13291	67	61
3.	933	P04217	**Alpha-1B-glycoprotein**	A1BG_HUMAN	5.58	54809	36	57
4.	227	P02768	**Serum albumin**	ALBU_HUMAN	5.92	71317	35	80
5.	210	Q6ZMW3	**Echinoderm microtubule-associated protein-like 6**	EMAL6_HUMAN	7.71	220270	14	62
6.	983	P02766	**Transthyretin**	TTHY_HUMAN	5.52	15991	59	60
7.	253	P01833	**Polymeric immunoglobulin receptor**	PIGR_HUMAN	5.58	84429	36	60
8.	237	P01833	**Polymeric immunoglobulin receptor**	PIGR_HUMAN	5.58	84429	31	82
9.	162	P03952	**Plasma kallikrein**	KLKB1_HUMAN	8.6	73433	17	63
10.	258	P04217	**Alpha-1B-glycoprotein**	A1BG_HUMAN	5.58	54809	36	57
11.	166	P00450	**Ceruloplasmin**	CERU_HUMAN	5.44	122983	14	58

12.	799	Q6U7Q0	**Zinc finger protein 322**	ZN322_HUMAN	8.98	48563	43	57
13.	163	Q96PF1	**Protein-glutamine gamma-glutamyl transferase Z**	TGM7_HUMAN	6.54	80575	16	63
14.	588	Q9UQ35	**Serine/arginine repetitive matrix protein 2**	SRRM2_HUMAN	12.05	300179	14	82
15.	593	O00443	**Phosphatidylinositol 4-phosphate 3-kinase C2 domain-containing subunit alpha**	P3C2A_HUMAN	8.25	192156	11	58
16.	318	P02768	**Serum albumin**	ALBU_HUMAN	5.92	71317	48	167
17.	618	Q15257	**Serine/threonine-protein phosphatase 2A activator**	PTPA_HUMAN	5.63	41098	26	57
18.	526	Q16769	**Glutaminyl-peptide cyclotransferase**	QPCT_HUMAN	6.12	40965	41	71
19.	215	Q6FIF0	**AN1-type zinc finger protein 6**	ZFAN6_HUMAN	6.87	23168	27	59
20.	266	Q92878	**DNA repair protein RAD50**	RAD50_HUMAN	6.48	154823	14	58
21.	584	Q9BUB4	**tRNA-specific adenosine deaminase 1**	ADAT1_HUMAN	9.20	56156	22	57
22.	882	P29508	**SERPINB3**	SPB3_HUMAN	6.35	44594	32	85
23.	765	Q9UKF7	**Cytoplasmic phosphatidylinositol transfer protein 1**	PITC1_HUMAN	5.99	38691	32	57
24.	902	Q16769	**Glutaminyl-peptide cyclotransferase**	QPCT_HUMAN	6.12	40965	44	80
25.	281	P01833	**Polymeric immunoglobulin receptor**	PIGR_HUMAN	5.58	84429	32	91
26.	998	Q8N3U1	**Putative uncharacterized protein LOC400692**	YS014_HUMAN	5.52	12915	46	64

27.	506	Q4V348	**Zinc finger protein 658B**	Z658B_HUMAN	8.9	97349	24	58
28.	655	O00560	**Syntenin-1**	SDCB1_HUMAN	7.05	32595	36	60
29.	1032	Q9H299	**SH3 domain-binding glutamic acid-rich-like protein 3**	SH3L3_HUMAN	4.82	10488	81	78
30.	959	P13645	**Keratin, type I cytoskeletal 10**	K1C10_HUMAN	5.13	59020	26	72
31.	770	P01833	**Polymeric immunoglobulin receptor**	PIGR_HUMAN	5.58	84429	40	138
32.	498	P04746	**Pancreatic alpha-amylase**	AMYP_HUMAN	6.6	58354	55	99

[a] Protein accession number for SWISSPROT Database.
[b] Theoretical isoelectric point.
[c] Theoretical relative mass.
[d] MASCOT score
